# EpCAM Is Essential to Maintaining the Immune Homeostasis of Intestines *via* Keeping the Expression of pIgR in the Intestinal Epithelium of Mice

**DOI:** 10.3389/fimmu.2022.843378

**Published:** 2022-04-13

**Authors:** Zili Lei, Wanwan Liu, Ya Nie, Yanhong Yang, Guibin Chen, Li Huang, Huijuan Wu, Yuting Lei, Lei Chen, Qing Hu, Hedong Rong, Siping Yu, Qi Song, Fengxue Tong, Jiao Guo

**Affiliations:** ^1^ Guangdong Metabolic Diseases Research Center of Integrated Chinese and Western Medicine, Key Laboratory of Glucolipid Metabolic Disorder, Ministry of Education of China, Guangdong Pharmaceutical University, Guangzhou, China; ^2^ School of Traditional Chinese Medicine, Guangdong Pharmaceutical University, Guangzhou Higher Education Mega Center, Guangzhou, China; ^3^ The First Affiliated Hospital, School of Clinical Medicine, Guangdong Pharmaceutical University, Guangzhou, China

**Keywords:** EpCAM, intestines, immune homeostasis, inflammation, pIgR

## Abstract

EpCAM deficiency causes congenital tufting enteropathy (CTE) which is considered as one kinds of very early onset inflammatory bowel disease (IBD). However, functions of EpCAM on regulating the immunity of intestines are still unclear. To study the mechanism of EpCAM on maintaining the intestinal immune homeostasis, the intestines of WT and EpCAM^-/-^ mice at E18.5, P0 and P3 stages were collected for morphological, histological and gene expression tests. Serious inflammation was detected in the small intestines of P3 EpCAM^-/-^ mice. Compared to WT mice, genes related to inflammatory factors and immunity cells, including TNFα, IL-1β, IL-6, IL-8rb, MIP2, MCP1, Ly6d and Ly6g, were all significantly upregulated and the expression of intestinal abundance matrix metalloproteinases (MMPs) was also significantly increased in the intestines of EpCAM^-/-^ mice at E18.5, P0 and P3 stages. Signals of p38, ERK1/2 and JNK were hyper-activated in the intestines of EpCAM^-/-^ mice. The expression of pIgR was significantly decreased and the expression and activation of transcriptional factors which promote the expression of pIgR were also reduced in the intestines of EpCAM^-/-^ mice compared to WT controls. In conclusion, EpCAM could maintain the immune homeostasis of intestines *via* keeping the expression of pIgR in the intestinal epithelium.

## Introduction

EpCAM is highly expressed in the intestinal epithelium, and the mutation of loss of function of EpCAM is associated with congenital tufting enteropathy (CTE) (1–4). CTE causes lethal diarrhea in newborns, and it is considered as one kinds of very early onset inflammatory bowel disease (5, 6). Previous studies demonstrated that impaired tight junctions in the intestinal epithelium of EpCAM mutant mice and patients was one of the important mechanisms of the severe diarrhea of them (7, 8). However, the inflammatory situation in EpCAM mutant mice and patients is still controversial. In some early clinical studies, histological results only showed mild to no inflammatory change in intestines of CTE patients (9). However, some researchers found significant increased plasma cells, eosinophils and lymphocytes in both small and large intestines of all the 8 CTE patients that they studied (10). Several reports confirmed that Claudin-7 was nearly lost at the protein level in the intestinal epithelium of EpCAM deficient mice and patients (2, 7, 8, 11, 12), and knockout of Claudin-7 caused inflammation in the intestines of mice (13–15). One previous study reported the inflammatory infiltration in intestines of EpCAM^-/-^ mice at postnatal day 4 (P4) (8). Hence, it is still essential to analyze the inflammatory situation and related mechanisms in EpCAM mutant mice and patients.

The dysregulation of intestinal immune homeostasis is one of the important mechanisms on the occurrence of inflammation in the mammalian gut (16, 17). The specialized innate and adaptive immune components are tightly regulating the immune response to various challenges in the intestines (18). Among these immune components, immunoglobulin A (IgA) and IgM are the first specific immunological defenses against infection in the intestinal lumen (19). IgA and IgM are produced by plasma cells under the intestinal epithelium (20), and are transported across the intestinal epithelial cells (IECs) by the polymeric immunoglobulin receptor (pIgR) localized at the basolateral surface of IECs (21). The transepithelial transport of IgA was found to be severely blocked in pIgR^-/-^ mice (22). Recent study reported that pIgR^-/-^ mice showed delayed growth and low-grade gut inflammation (23). Several transcriptional factors function to regulate the transcription of pIgR under the control of multiple signal pathways. IRF1 induces the transcription of pIgR through binding to the cognate element in intron 1 of it, and STAT1 mediates the function of IFNγ on the *de novo* transcription of IRF1 (24, 25). STAT6 binds to the cognate element in intron 1 of pIgR gene and mediates the function of IL-4 on regulating the transcription of pIgR (24, 25). NF-κB also can induce the transcription of pIgR *via* binding to the element in the intron 1 of it (24, 25). EpCAM is also enriched at the basolateral membrane of IECs, and it interacts with components of several signaling pathways on membrane of cells (2, 26). However, the relationship between EpCAM and pIgR is still unclear.

In the present study, the inflammatory situation in intestines of EpCAM^-/-^ mice was confirmed at both tissue and molecular levels. The downregulation of pIgR and transcriptional factors which control the expression of pIgR in the intestinal epithelium might be the important mechanism of the occurrence of inflammation in the intestines of EpCAM^-/-^ mice. Our discovery encouraged the exploration for mechanisms on maintaining the immune homeostasis of the mammalian intestines.

## Materials and Methods

### Mice

All animal experimental procedures were approved by the Experimental Animal Ethics Committee of Guangdong Pharmaceutical University. WT and EpCAM^-/-^ embryos and pups were got from mating the EpCAM^+/-^ parental mice previously generated (27). EpCAM^+/-^ mice were maintained on C57BL/6 background. Mice were housed in the SPF mouse facility, at 25°C, 12hr light-dark cycle, 60-65% humidity, with free access to water and food.

### Hematoxylin and Eosin (H&E) Staining

The duodenum, jejunum, ileum and colon which were collected from mouse embryos and pups were fixed in 4% paraformaldehyde in PBS at 4°C for overnight and then were embedded in paraffin for H&E staining. The 4-µm-thick paraffin sections were stained with hematoxylin (H9627, Sigma-Aldrich) for 3 min and then followed with eosin (E4009, Sigma-Aldrich) for 20 sec at room temperature. The images of the sections were taken with the PerkinElmer Automated Quantitative Pathology System (PerkinElmer, Inc.).

### Immunostaining

For immunostaining, intestinal tissues were fixed in 4% paraformaldehyde in PBS at 4°C for overnight and then were embedded in optimal cutting temperature compound (OCT) (Sakura Finetek). 7-µm-thick frozen sections were boiled in 50 mM sodium citrate buffer solution (pH=6.0) for antigen retrieval, then were blocked using 1% BSA in PBS at room temperature for 1 hour. Subsequently, the sections were incubated with primary antibodies in PBS with 1% BSA at 4°C for overnight, and then incubated with secondary antibodies in PBS with 1% BSA at room temperature for 1 hour. The primary antibodies included rabbit anti-EpCAM (1:200; ab71916; Abcam), rabbit anti-mouse IgA secondary antibody (1:1000; NB7506; Novus) and rabbit anti-Claudin-7 (1:200; 34-9100; Thermo Fisher Scientific, Inc.). Immunohistochemical analysis was performed with biotin-conjugated goat-anti-rabbit secondary antibody (JAC-111-065-144, Jackson ImmunoResearch), HRP-ABC complex (CA 94010, VECTASTAIN) and DAB (LF778, DOJINDO Laboratories), and immunofluorescence analysis was performed with Alex Fluor 488-labeled secondary antibodies (Invitrogen). The immunostaining images were observed using the PerkinElmer Automated Quantitative Pathology System.

### qRT-PCR

Total RNA was extracted from each small or large intestinal tissue from embryos and pups using Trizol reagent (Invitrogen; Thermo Fisher Scientific, Inc.), and then was subjected to reverse transcription using the PrimeScript™ RT Reagent kit (Takara Bio, Inc.) at 37°C for 15 min and then 85°C for 5 sec. qPCR was performed using the SYBR Premix Ex Taq kit (Takara Bio, Inc.) in the LightCycler 480II System (Roche, Inc.). The processes of cycling were as followed: 95°C for 30 sec; and then 40 cycles of 95°C for 5 sec, 60°C for 20 sec and 65°C for 15 sec. GAPDH was used as the reference. All primers used for qPCR in the present study were listed in **Table S1**.

### Western Blot

The small or large intestinal tissues from embryos and pups were lysed in Radio-Immunoprecipitation Assay lysis buffer (Dalian Meilun Biotechnology co., Ltd.), and then were centrifuged at 13,680 x g, at 4°C, for 30 min, and then the supernatant was harvested. Protein concentration was measured using BCA kit (P0011, Beyotime). After that, equal amounts of protein were separated using the SDS-PAGE (8-12% gel), and subsequently were transferred to PVDF membrane. The PVDF membrane was blocked with 5% skimmed milk in TBST buffer at room temperature for 1 hour, then was incubated with primary antibodies at 4°C for overnight, and then was incubated with HRP (horseradish peroxidase)-labeled secondary antibodies, the signals were detected using enhanced chemiluminescence reagent. The quantification of WB bands was analyzed using the ImageJ software (version 1.53a). The primary and secondary antibodies used for WB were listed in **Table S2**.

### Statistical Analysis

Statistical analysis was determined *via* the SPSS software (version 23.0; IBM Corp.). Mean ± SEM was used to express data. Mann–Whitney U test was conducted to compare difference between two groups, and the P-value <0.05 was considered to be significant.

## Results

### EpCAM Deficiency Caused Serious Intestinal Inflammation in Postnatal Mice

**Figure 1:**
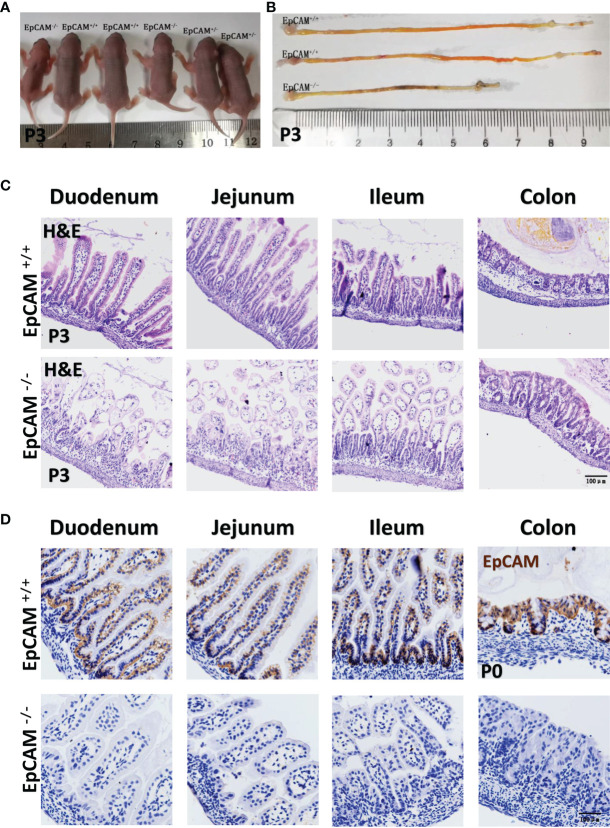
EpCAM deficiency caused inflammation in the intestines of postnatal mice. **(A)** A littermate of P3 pups from one pair of EpCAM^+/-^ parental mice. **(B)** The images of intestines from WT and EpCAM^-/-^ pups at P3 stage. **(C)** Images of H&E staining of duodenum, jejunum, ileum and colon from WT and EpCAM^-/-^ pups at P3 stage. **(D)** Images of the immunohistochemical staining of EpCAM in the sections of duodenum, jejunum, ileum and colon from WT and EpCAM^-/-^ pups at P0 stage.

To test if the mutation of EpCAM could induce inflammation in the intestines, embryos and pups at E18.5, P0 and p3 stages from EpCAM^+/-^ parental mice were harvested and both WT and EpCAM^-/-^ individuals were selected for experiments. The EpCAM^-/-^ pups at P0 stage still looked no significant difference with the WT and heterozygotes pups from the same littermates (**Supplementary Figure 1A**). However, the body size of EpCAM^-/-^ pups at P3 stage was significantly smaller than that of WT and EpCAM^+/-^ pups from the same littermates (**Figure 1A**). The morphology of intestines from the P0 EpCAM^-/-^ pups was still normal (**Supplementary Figure 1B**). The length of small intestines from EpCAM^-/-^ pups at P0 stage was slightly but significantly shorter than that from WT pups (**Supplementary Figure 1C**). The intestines from P3 EpCAM^-/-^ pups were significantly shorter than that from the WT pups at the same stages, especially for the small intestines; and blood could be found in the lumen of intestines of the P3 EpCAM^-/-^ pups (**Figure 1B** and **Supplementary Figure 1C**). The tufting of villi could be observed in the intestines of EpCAM^-/-^ pups at both P0 and P3 stages (**Figure 1C** and **Supplementary Figure 1D**). Proteins of EpCAM could not be detected in the intestinal epithelium of EpCAM^-/-^ pups (**Figure 1D**). The Claudin-7 proteins also could not be detected in the intestines of EpCAM^-/-^ embryos (**Supplementary Figure 1E**). These results demonstrated that the EpCAM^-/-^ mice in the present study could be used as animal models of CTE.

The H&E staining results showed that the small intestines of EpCAM^-/-^ pups were slightly damaged at P0 stage and numbers of inflammatory cells were also slightly increased in the small intestines of EpCAM^-/-^ pups at this stage, especially for the jejunum sections (**Supplementary Figure 1D**). At P3 stage, the small intestines of EpCAM^-/-^ pups were seriously damaged and numbers of infiltrated inflammatory cells were also significantly increased in these sections (**Figure 1C**). However, colons of EpCAM^-/-^ pups still looked normal at both P0 and P3 stages (**Figure 1C** and **Supplementary Figure 1D**). These results indicated the occurrence of serious inflammation in small intestines of postnatal EpCAM^-/-^ mice.

### EpCAM Deficiency Induced the Upregulation of Genes Related to Inflammatory Factors and Immunity Cells in the Intestines of Mice

**Figure 2:**
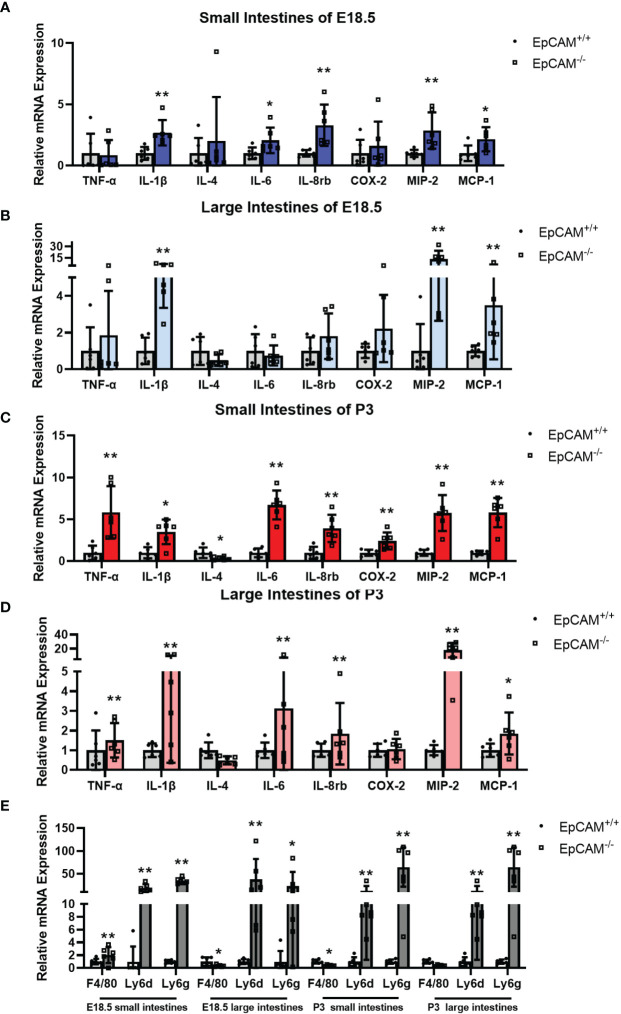
EpCAM deficiency induced the upregulation of genes related to inflammatory factors and the infiltration of inflammatory cells in the intestines of mice. **(A, B)** Graphs showed the relative mRNA expression levels of TNF-α, IL-1β, IL-4, IL-6, IL-8rb, COX-2, MIP-2 and MCP-1 in the **(A)** small and **(B)** large intestines from WT and EpCAM^-/-^ embryos at E18.5 stage (n=6). **(C, D)** Graphs showed the relative mRNA expression levels of TNF-α, IL-1β, IL-4, IL-6, IL-8rb, COX-2, MIP-2 and MCP-1 in the **(C)** small and **(D)** large intestines from WT and EpCAM^-/-^ pups at P3 stage (n=6). **(E)** qPCR results of F4/80, Ly6d and Ly6g from the small and large intestines of WT and EpCAM^-/-^ mice at E18.5 and P3 stages, respectively (n=6). *p < 0.05, **p < 0.01, as determined by Mann–Whitney U test. All error bars represent SDs.

To confirm the occurrence of inflammation in the intestines of EpCAM^-/-^ mice, the mRNA levels of genes related to inflammatory factors and immunity cells were checked. At E18.5 stage, the mRNA levels of IL-1β, IL-6, IL-8rb, MIP-2 and MCP-1 were all significantly increased to 2-3 folds in the small intestines of EpCAM^-/-^ embryos compared to the WT mice (**Figure 2A**). The mRNA level of F4/80, which is the cell surface marker of macrophages (15, 28), increased to around 2 folds and the transcriptional level of the marker of neutrophils Ly6g (15) was increased to around 30 folds in the small intestines of EpCAM^-/-^ embryos compared to WT embryonic small intestines, and the mRNA level of Ly6d, which is one of the novel inflammation markers (29), was also increased nearly 30 folds in the small intestines of EpCAM^-/-^ mice (**Figure 2E**). In the large intestines of E18.5 EpCAM^-/-^ mice, the mRNA levels of IL-1β, MIP2 and MCP-1were all significantly higher than WT embryos (**Figure 2B**). The mRNA levels of Ly6d and Ly6g were also increased nearly 50 folds in the large intestines of EpCAM^-/-^ embryos at E18.5 stage, although the mRNA level of F4/80 was significantly decreased in the large intestines of EpCAM^-/-^ E18.5 embryos (**Figure 2E**). At P3 stage, the mRNA levels of TNFα, IL-1β, IL-6, IL-8rb, COX-2, MIP-2 and MCP-1 were all significantly increased from 2 to nearly 7 folds in the small intestines of EpCAM^-/-^ mice (**Figure 2C**). Although the mRNA level of F4/80 was significantly decreased in the small intestines of P3 EpCAM^-/-^ mice, the mRNA levels of Ly6d and Ly6g were all significantly increased in small intestines of EpCAM^-/-^ pups at P3 stages compared to P3 WT pups (**Figure 2E**). In large intestines of P3 pups, the mRNA levels of TNFα, IL-1β, IL-6, IL-8rb, MIP-2 and MCP-1 were all significantly increased (**Figure 2D**). The mRNA levels of Ly6d and Ly6g in the large intestines of P3 EpCAM^-/-^ pups were 5-fold and nearly 100-fold higher than those of WT pups, respectively; although the mRNA level of F4/80 had no significant change in the large intestines of EpCAM^-/-^ pups at P3 stage (**Figure 2E**). The changes of mRNA levels of these genes in the intestines of EpCAM^-/-^ mice at P0 stage were shown in **Supplementary Figure 2**. These results confirmed that EpCAM deficiency induced severe inflammation in the intestines of mice.

### EpCAM Deficiency Caused the Hyper-Activation of MAPK Signal Pathways in the Intestines of Mice

**Figure 3:**
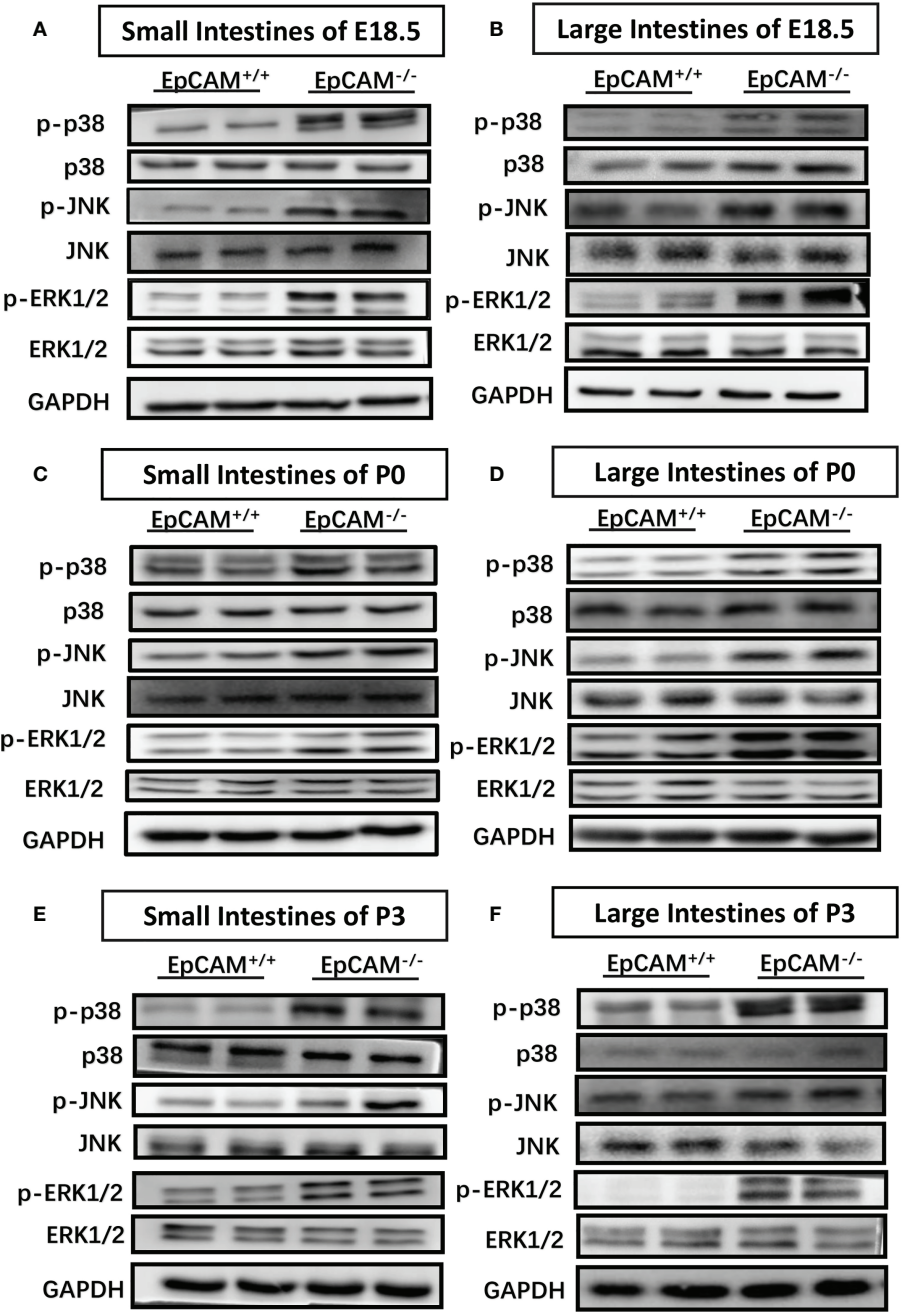
EpCAM deficiency caused the hyper-activation of inflammation related MAPK signal pathways in the intestines of mice. **(A, B)** Western blot results of p-p38, p38, p-JNK, JNK, p-ERK1/2 and ERK1/2 in **(A)** the small intestines and **(B)** large intestines from WT and EpCAM^-/-^ embryos at E18.5 stage (n=3). **(C, D)**. Western blot results of p-p38, p38, p-JNK, JNK, p-ERK1/2 and ERK1/2 in **(C)** the small intestines and **(D)** large intestines from WT and EpCAM^-/-^ pups at P0 stage (n=3). **(E, F)** Western blot results of p-p38, p38, p-JNK, JNK, p-ERK1/2 and ERK1/2 in **(E)** the small intestines and **(F)** large intestines from WT and EpCAM^-/-^ pups at P3 stage (n=3).

Signal pathways related to inflammatory response were subsequently tested for further confirm of the occurrence of inflammation in the intestines of EpCAM^-/-^ mice at molecular levels. The phosphorylation levels of JNK were significantly elevated in small intestines of EpCAM^-/-^ mice at E18.5, P0 and P3 stages compared to the WT mice, although there was no significant difference of the total protein of JNK in the small intestines between WT and EpCAM^-/-^ mice at these stages (**Figures 3A, C, E**). The p-JNK levels were also significantly increased in the large intestines of EpCAM^-/-^ mice at both E18.5 and P0 stages compared to WT mice at the same stages, and the total protein levels of JNK were similar between EpCAM^-/-^ and WT mice at these two stages (**Figures 3B, D**). However, the total protein level of JNK was reduced in the large intestines of EpCAM^-/-^ mice compared to WT mice at P3 stage, and the level of p-JNK was only slightly increased in the large intestines of P3 EpCAM^-/-^ mice compared to WT pups (**Figure 3F**). The levels of phosphorylated p38 were elevated in both small and large intestines of EpCAM^-/-^ mice at E18.5 stage compared to WT embryos, and the total protein had no significant change in small intestines of EpCAM^-/-^ mice although it was increased in the large intestines of EpCAM^-/-^ mice at E18.5 stage (**Figures 3A, B**). The phosphorylation levels of p38 were significantly elevated in both small and large intestines of EpCAM^-/-^ pups at P0 and P3 stages compared to WT pups, and the levels of total protein of p38 showed no significant change in both small and large intestines of EpCAM^-/-^ pups at these stages (**Figures 3C–F**). The phosphorylation levels of ERK1/2 were all significantly increased in both small and large intestines of EpCAM^-/-^ mice at E18.5, P0 and P3 stages compared to WT mice at same stages, and the levels of total protein of ERK1/2 were similar in the intestines of both WT and EpCAM^-/-^ mice at these stages (**Figures 3A–F**). These results indicated that the inflammation related MAPK signals were hyper-activated in both small and large intestines of EpCAM^-/-^ mice at E18.5, P0 and P3 stages.

### EpCAM Deficiency Upregulated the Expression of Genes for Matrix Metalloproteinases in the Intestines of Mice

**Figure 4:**
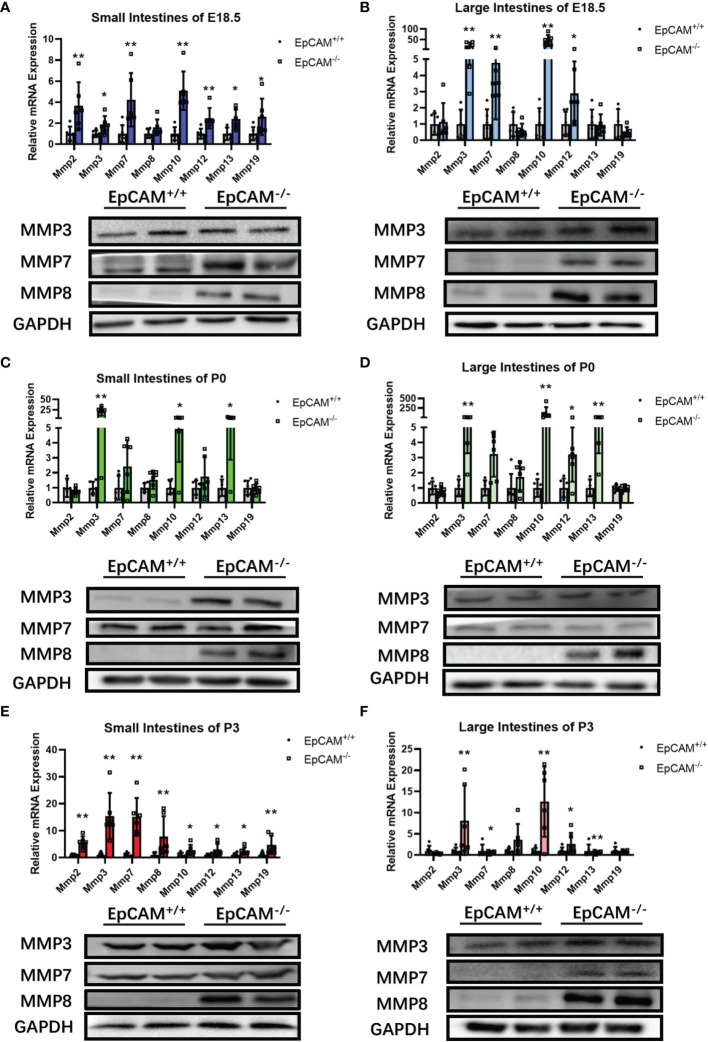
EpCAM deficiency induced the overexpression of genes for matrix metalloproteinases in the intestines of mice. **(A, B)** The relative mRNA expression levels of Mmp2, 3, 8, 10, 12, 13 and 19 (n=6) and the Western blot results of MMP3, 7 and 8 (n=3) in the **(A)** small and **(B)** large intestines from WT and EpCAM^-/-^ embryos at E18.5 stage. **(C, D)** The relative mRNA expression levels of Mmp2, 3, 8, 10, 12, 13 and 19 (n=6) and the Western blot results of MMP3, 7 and 8 (n=3) in the **(C)** small and **(D)** large intestines from WT and EpCAM^-/-^ pups at P0 stage. **(E, F)** The relative mRNA expression levels of Mmp2, 3, 8, 10, 12, 13 (n=6) and 19 and the Western blot results of MMP3, 7 and 8 in the **(E)** small and **(F)** large intestines from WT and EpCAM^-/-^ pups at P3 stage (n=3). *p < 0.05, **p < 0.01, as determined by Mann–Whitney U test. All error bars represent SDs.

To study the molecular mechanism on inducing the serious damage of the intestines of EpCAM^-/-^ mice, the expression levels of intestinal abundant matrix metalloproteinases (MMPs) were checked. Although the mRNA level of MMP8 was only significantly higher in the small intestines of P3 EpCAM^-/-^ pups than that of WT mice, the protein levels of it were clearly elevated in both small and large intestines of EpCAM^-/-^ mice at E18.5, P0 and P3 stages compared to WT mice (**Figures 4A–F**). Both mRNA and protein levels of MMP7 were all clearly increased in the small and large intestines of EpCAM^-/-^ mice at E18.5 and P3 stages compared to the WT mice at these two stages (**Figures 4A, B, E, F**). The mRNA levels of MMP7 were also significantly increased in both small and large intestines of P0 EpCAM^-/-^ pups compared to WT pups, but the protein levels of MMP7 showed no significant difference in both small and large intestines of WT and EpCAM^-/-^ pups at P0 stage (**Figures 4C, D**). The mRNA level of MMP3 was also increased from 3 to nearly 100 folds in the intestines of EpCAM^-/-^ mice at E18.5, P0 and P3 stages compared to WT mice, and proteins of MMP3 elevated in small intestines of P0 EpCAM^-/-^ pups and in both small and large intestines of P3 EpCAM^-/-^ pups compared to WT mice (**Figures 4A–F**). The mRNA levels of MMP10, 12, 13 and 19 were also significantly upregulated in most parts of intestines from EpCAM^-/-^ mice (**Figures 4A–F**). These results indicated that the increase of MMPs might be one of the important reasons for the damage of intestines from EpCAM^-/-^ pups.

### EpCAM Deficiency Downregulated the Expression of pIgR in the Intestines of Mice

In order to analyze the mechanism on causing the inflammation in the intestines of EpCAM^-/-^ mice, the expression levels of IgA, IgM and pIgR related genes were first checked. The mRNA levels of Igha and Ighm were tested, and there was no significant difference of Igha in small intestines between WT and EpCAM^-/-^ mice at E18.5 and P0 stages (**Supplementary Figure 3A**). However, the transcriptional level of Igha was significantly reduced in the small intestines of P3 EpCAM^-/-^ mice (**Supplementary Figure 3A**). The mRNA level of Igha showed no significant change in large intestines of EpCAM^-/-^ mice at E18.5 and P3 stages, but it was significantly reduced in the large intestines of EpCAM^-/-^ mice at P0 stage (**Supplementary Figure 3A**). The transcriptional levels of Ighm had no significant change in both small and large intestines of EpCAM^-/-^ mice at E18.5 and P0 stages, but they were significantly reduced in both small and large intestines of EpCAM^-/-^ mice at P3 stage compared to WT pups (**Supplementary Figure 3B**). The immunostaining results showed no clear difference of the protein levels of IgA in both small and large intestines between WT and EpCAM^-/-^ mice at E18.5 and P3 stages (**Supplementary Figure 3C**).

**Figure 5:**
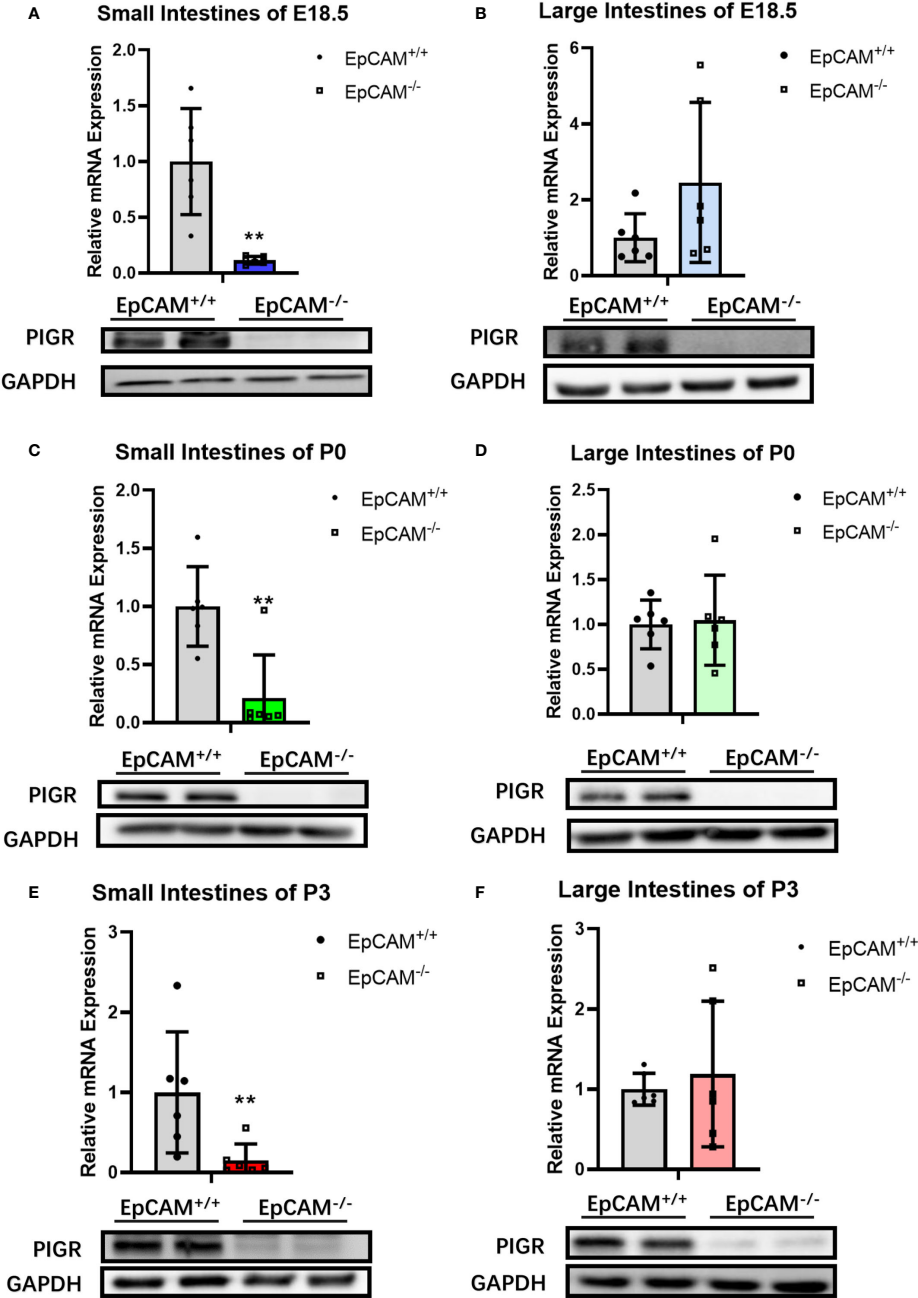
EpCAM deficiency caused the downregulation of pIgR in the intestines of mice. **(A)** The mRNA (n=6) and protein (n=3) expression levels of pIgR in the small intestines from WT and EpCAM^-/-^ embryos at E18.5 stage. **(B)** The mRNA (n=6) and protein (n=3) expression levels of pIgR in the large intestines from WT and EpCAM^-/-^ embryos at E18.5 stage. **(C)** The mRNA (n=6) and protein (n=3) expression levels of pIgR in the small intestines from WT and EpCAM^-/-^ pups at P0 stage. **(D)** The mRNA (n=6) and protein (n=3) expression levels of pIgR in the large intestines from WT and EpCAM^-/-^ pups at P0 stage. **(E)** The mRNA (n=6) and protein (n=3) expression levels of pIgR in the small intestines from WT and EpCAM^-/-^ pups at P3 stage. **(F)** The mRNA (n=6) and protein (n=3) expression levels of pIgR in the large intestines from WT and EpCAM^-/-^ pups at P3 stage. **p < 0.01, as determined by Mann–Whitney U test. All error bars represent SDs.

The proteins of pIgR were hard to be detected in both small and large intestines of EpCAM^-/-^ mice at E18.5, P0 and P3 stages, although they are highly expressed in the intestines of WT mice (**Figures 5A–F**). The mRNA levels of pIgR were also significantly lower in the small intestines of EpCAM^-/-^ mice at E18.5, P0 and P3 stages than that of WT mice, but there was no significant change of the transcriptional expression of pIgR in the large intestines of EpCAM^-/-^ mice at these stages (**Figures 5A–F**). These results demonstrated that the reduction of pIgR might be one of the important mechanisms of the occurrence of inflammation in the intestines of EpCAM^-/-^ mice.

### EpCAM Deficiency Downregulated the Transcriptional Factors Which Regulated the Expression of pIgR in the Intestinal Epithelium of Mice

**Figure 6:**
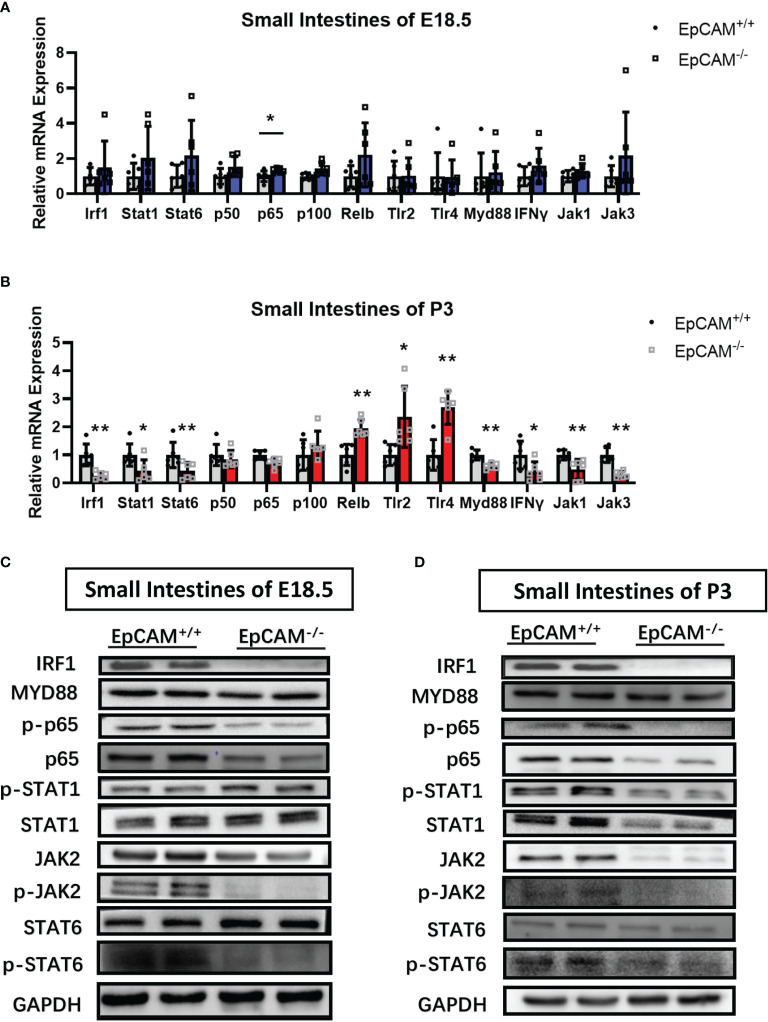
EpCAM deficiency caused the changes of signaling pathways which regulate the expression of pIgR in the epithelial cells from the small intestines of mice. **(A, B)** Graphs showed the qPCR results of Irf1, Stat1, Stat6, p50, p65, p100, Relb, Tlr2, Tlr4, Myd88, IFNγ, Jak1 and Jak3 in the small intestines from WT and EpCAM^-/-^ mice at **(A)** E18.5 and **(B)** P3 stages, respectively (n=6). **(C, D)** Western blot results of IRF1, p-STAT1, STAT1, p-JAK2, JAK2, MYD88, p-p65, p65, p-STAT6 and STAT6 in the small intestines from WT and EpCAM^-/-^ mice at **(C)** E18.5 and **(D)** P3 stages, respectively (n=3). *p < 0.05, **p < 0.01, as determined by Mann–Whitney U test. All error bars represent SDs.

The transcriptional levels of most genes related to signaling pathways which regulate the transcription of pIgR, including TLR2, TLR4, Myd88, Stat1, Stat6, IFNγ, IRF1, p50, p100, JAK1 and JAK3, were not significantly changed in both small and large intestines of EpCAM^-/-^ mice at E18.5 stage compared to WT mice, although the mRNA of p65 was significantly increased in the small intestines and the mRNA of Relb and TLR4 was also significantly increased around 3 folds in the large intestines of EpCAM^-/-^ mice at E18.5 stage (**Figure 6A** and **Supplementary Figure 4A**). However, at P3 stage, the mRNA levels of IRF1, Stat1, Stat6, Myd88, IFNγ, JAK1 and JAK3 were all significantly reduced in the small intestines of EpCAM^-/-^ mice compared to WT pups, but the mRNA levels of Relb, TLR2 and TLR4 were 2-3 folds higher in the small intestines of EpCAM^-/-^ mice than that of WT pups (**Figure 6B**). The mRNA levels of most of these genes showed no significant change in the large intestines of P3 EpCAM^-/-^ mice except the slightly significant increase of TLR4 (**Supplementary Figure 4B**). The protein levels of IRF1 were significantly reduced in both small and large intestines of EpCAM^-/-^ mice at E18.5 and P3 stages, and they were even hard to be detected in the small intestines of EpCAM^-/-^ mice (**Figures 6C, D** and **Supplementary Figures 4C, D**). Both the activated and total proteins of STAT1were decreased in the small intestines of EpCAM^-/-^ P3 pups and in the large intestines of EpCAM^-/-^ E18.5 embryos, although there were no significant differences of them in the E18.5 small intestines and P3 large intestines between WT and EpCAM^-/-^ mice (**Figures 6C, D** and **Supplementary Figures 4C, D**).

The levels of phosphorylated STAT6 were significantly reduced in the small intestines of EpCAM^-/-^ mice at E18.5 and P3 stages, although the total protein of STAT6 showed no significant change in these small intestines (**Figures 6C, D**). However, both the total and phosphorylated proteins of STAT6 were all reduced in the large intestines of EpCAM^-/-^ mice at E18.5 and P3 stages (**Supplementary Figures 4C, D**). Both the activated and total proteins of p65 were all clearly reduced in small and large intestines of EpCAM^-/-^ mice at E18.5 and P3 stages (**Figures 6C, D** and **Supplementary Figures 4C, D**). The changes of the expression of these genes in EpCAM^-/-^ mice at P0 stage were shown in **Supplementary Figure 5**.

The above results demonstrated that the expression and activation of transcriptional factors which induce the transcription of pIgR, including IRF1, Stat6 and NF-κB, were all downregulated in the intestinal epithelium of EpCAM^-/-^ mice. The downregulation of these transcriptional factors might be the important reasons for the reduction of the expression of pIgR in the intestines of EpCM^-/-^ mice.

## Discussion

Herein, we uncovered a new role of EpCAM in maintaining the intestinal immune homeostasis. We first confirmed the occurrence of serious inflammation in the intestines of postnatal EpCAM^-/-^ mice through morphological and histological methods. Then, qPCR results showed the significant increase of genes related to inflammatory factors and immunity cells in both small and large intestines of EpCAM^-/-^ mice. After that, Western blot results demonstrated the hyper-activation of inflammation related MAPK signal pathways in the intestines of EpCAM^-/-^ mice. Then, we found that several intestinal abundant MMPs were upregulated in the intestines of EpCAM^-/-^ mice. Finally, we discovered that pIgR and transcriptional factors which induce the transcription of pIgR, including IRF1, STAT6 and NF-κB, were all downregulated in the intestines of EpCAM^-/-^ mice. These results indicated the function and mechanism of EpCAM on maintaining the immune homeostasis of intestinal epithelium.

Because of the rarity and severity of CTE (3), it is difficult to analyze the inflammatory situation in each segments of intestines from patients at various stages. Most previous studies of EpCAM^-/-^ mice focused on cell-cell junctions in the intestinal epithelium and detected the loss of Claudin-7 proteins in the intestinal tissue of them (7, 8). The Claudin-7 proteins were also nearly lost in the intestines of EpCAM^-/-^ mice in the present study (**Supplementary Figure 1E**). The intestines of Claudin-7 knockout mice were still normal at P0 stage, but serious inflammation occurred in both small and large intestines within 3 to 5 days after birth (13). Previous study reported that the occurrence of edema and inflammatory infiltration in the small intestines of EpCAM^-/-^ pups at P4 stage, but the colon of these mice was unaffected (8). However, knockdown of EpCAM in the colon of mice also could increase the severity of dextran sulfate sodium salt (DSS)-induced murine inflammatory bowel disease (IBD) (30). The infiltration of inflammatory cells could be detected at P0 stage in the small intestines of the EpCAM^-/-^ mice in the present study, and the inflammation became very serious in the small intestines at P3 stage. Although the morphology of the colon in EpCAM^-/-^ mice still looked normal at P3 stage, the mRNA levels of the inflammatory cells, including Ly6d and Ly6g, were significantly increased indicating the infiltration of inflammatory cells in the colon tissues. The increase of mRNA levels of Ly6d and Ly6g in both small and large intestines of EpCAM^-/-^ mice at E18.5 stage firstly demonstrated the occurrence of inflammation in the intestines at the late embryonic stage. The mutant form of EpCAM protein was still expressed at much lower levels in the intestines of EpCAM^-/-^ mice generated in the previous report (8). However, the EpCAM protein was completely lost in the present EpCAM^-/-^ mice (**Figure 1D**). Therefore, the inflammation occurred very earlier in the intestines of the present EpCAM^-/-^ mice than the previous study. The loss of Claudin-7 protein in the intestinal epithelium of EpCAM^-/-^ mice might be one of the important reasons of the initiation of inflammation, but the mechanism of Claudin-7 on regulating the immune homeostasis of intestinal epithelium is not completely clear.

The hyper-activation of MAPKs had been found in the inflammatory intestinal tissues of patients and animal models. The increase of the phosphorylation of p38 MAPK was observed in intestinal samples from patients of both Crohn’s disease (CD) and ulcerative colitis (UC) (31). The increased levels of p-ERK1/2, p-p38 and p-JNK were also detected in DSS-induced UC intestinal tissues of mice (32). In present study, the hyper-activation of ERK1/2, p38 and JNK was found in both small and large intestines of EpCAM^-/-^ mice at E18.5, P0 and P3 stages (**Figure 3**), and the hyper-activation of these MAPKs confirmed the inflammatory situation in both small and large intestines of EpCAM^-/-^ mice which was consistent with the increase of the expression of genes related to inflammatory factors and markers of immunity cells in the intestines of these mice.

MMPs are produced in excess in inflamed intestinal tissues of IBD patients and causes mucosal degradation (33). Recent study showed that MMP12 mediated the degradation of basement membrane laminin and the transmigration across intestinal epithelial tight junctions of macrophages, and increased severity of DSS-induced colitis of mice (34). It was also found that the elevated MMP3 and MMP9 induced by serotonine might be associated with the serotonine-exacerbated DSS-induced colitis of mice (35), and the increased circulating MMP3 and MMP9 had been considered as biomarkers of the clinical activity of IBD (36, 37). In LPS-induced acute inflammation, the increased MMP7 showed pro-inflammatory effects in intestines of mice *via* activating macrophages and amplifying local inflammatory response (38). MMP8 was reported to exacerbate the intestinal ischemia-reperfusion injury of mice *via* increasing inflammation and reducing the expression of Claudin-3 (39). At present, the mRNA and protein levels of MMP3, MMP7 and MMP8 were all significantly increased in the intestines of EpCAM^-/-^ mice (**Figure 4**). We speculated that the increase of MMPs might be one of the important reasons of the degradation of the intestinal tissues and the increase of the infiltration of inflammatory cells in the intestines of EpCAM^-/-^ mice.

The PIGR gene was detected to be significantly affected by non-synonymous mutations in the intestinal epithelium of samples from patients with UC (40). It was reported that CD patients showed decreased median expression of PIGR in non-inflamed colonic mucosa and patients with UC also exhibited decreased expression of PIGR in colonic mucosa compared to the health (41). The expression of pIgR was reduced in the colonic epithelial cells of both DSS induced acute colitis and T-cell induced chronic colitis of mouse models (42). In the present study, the protein level of PIGR became very low in the intestines of EpCAM^-/-^ mice (**Figure 5**), and the transport of IgA and IgM into the lumen of intestines might be severely blocked. So, the reduction of PIGR in the intestinal epithelium of EpCAM^-/-^ mice might lead to the inflammatory response to stimulations in the intestinal lumen of these mice.

Multiple signaling pathways have been demonstrated to regulate the transcription of pIgR, including JAK-STAT and NF-κB signaling pathways (25). The interferon-stimulated response element (ISRE) in the exon 1 of PIGR gene in HT-29 human colon carcinoma cells binds IRF-1 following the stimulation of IFNγ to enhance the transcription of pIgR (43). It was confirmed that IRF-1 has important role in regulating the transcription of pIgR gene in intestinal epithelial cells both *in vivo* and *in vitro* stimulated by IFNγ (44, 45), and the mRNA level of pIgR was greatly reduced in the intestines and liver of IRF-1-deficient mice (44). IFNγ usually stimulates the transcription of IRF-1 through activating STAT1 in the intestinal epithelium (25, 46). Hence, the downregulation of STAT1 and IRF-1 in the intestines of EpCAM^-/-^ mice might be one of the important mechanisms of the reduction of the expression of pIgR in the intestinal epithelium of EpCAM^-/-^ mice. The activated STAT6 had been demonstrated to bind to a 554-bp IL-4 responsive enhancer in intron 1 of human PIGR gene in HT-29 cells to upregulate the expression of PIGR (47). It was reported that IL-4 mediated the transcription of pIgR *via* activating JAK1-STAT6 pathway in the intestines of mice (46, 48, 49). In present study, the levels of phosphorylated STAT6 were reduced in the intestines of EpCAM^-/-^ mice, and the transcriptional levels of IL-4, Jak1 and Stat6 also reduced in the intestines of these mice. The reduction of the IL-4/JAK1/STAT6 pathway was another mechanism of the decrease of the transcription of pIgR in the intestines of EpCAM^-/-^ mice.

Several TNFα-responsive regions which could bind with NF-κB had been identified in the promoter of PIGR gene in HT-29 cells including one site in intron 1 of this gene, and TNFα increased the expression of PIGR through the biding of NF-κB to these regions (50–52). The TLR3 and TLR4 signals had been found to stimulate the transcription of PIGR in HT-29 cells *via* NF-κB (53, 54), and the expression of pIgR was significantly reduced in colons of Myd88^-/-^ mice indicating the role of TLRs/MYD88/NF-κB pathway in regulating the transcription of pIgR *in vivo* (53). It was confirmed that upregulation of PIGR gene in HT-29 cells required both RelA-dependent classical and RelB-dependent alternative pathways of NF-κB (55). Currently, the levels of p65 and p-p65 were all reduced in the intestines of EpCAM^-/-^ mice indicating that the NF-κB signals became weak in the EpCAM^-/-^ intestines. Our previous work reported that p65 and p-p65 reduced in the colon of mice treated with high dose of LiCl and the expression of pIgR was also downregulated in the colon of these mice (28). The reduction of NF-κB signals might also be the mechanism of the downregulation of the expression of pIgR in the intestines of EpCAM^-/-^ mice.

The expression and activation of transcriptional factors, including STAT1, IRF1, STAT6 and NF-κB, were all reduced in both small and large intestines of EpCAM^-/-^ mice, and the expression of pIgR was significantly down-regulated in small intestines of EpCAM^-/-^ mice at both mRNA and protein levels. However, the mRNA of pIgR showed no significant change in the large intestines of EpCAM^-/-^ mice demonstrating that there might be some other transcriptional factors which could regulate the transcription of pIgR in the large intestines of mice. However, the protein level of PIGR was significantly downregulated in large intestines of EpCAM^-/-^ mice, demonstrating that EpCAM also could regulate the expression of pIgR at the post-transcriptional level in the large intestines of mice. The related mechanism of these regulations will be explored in our future work. Therefore, EpCAM might maintain the normal level of PIGR in the intestinal epithelium *via* interacting with multiple signal pathways in IECs.

**Figure 7:**
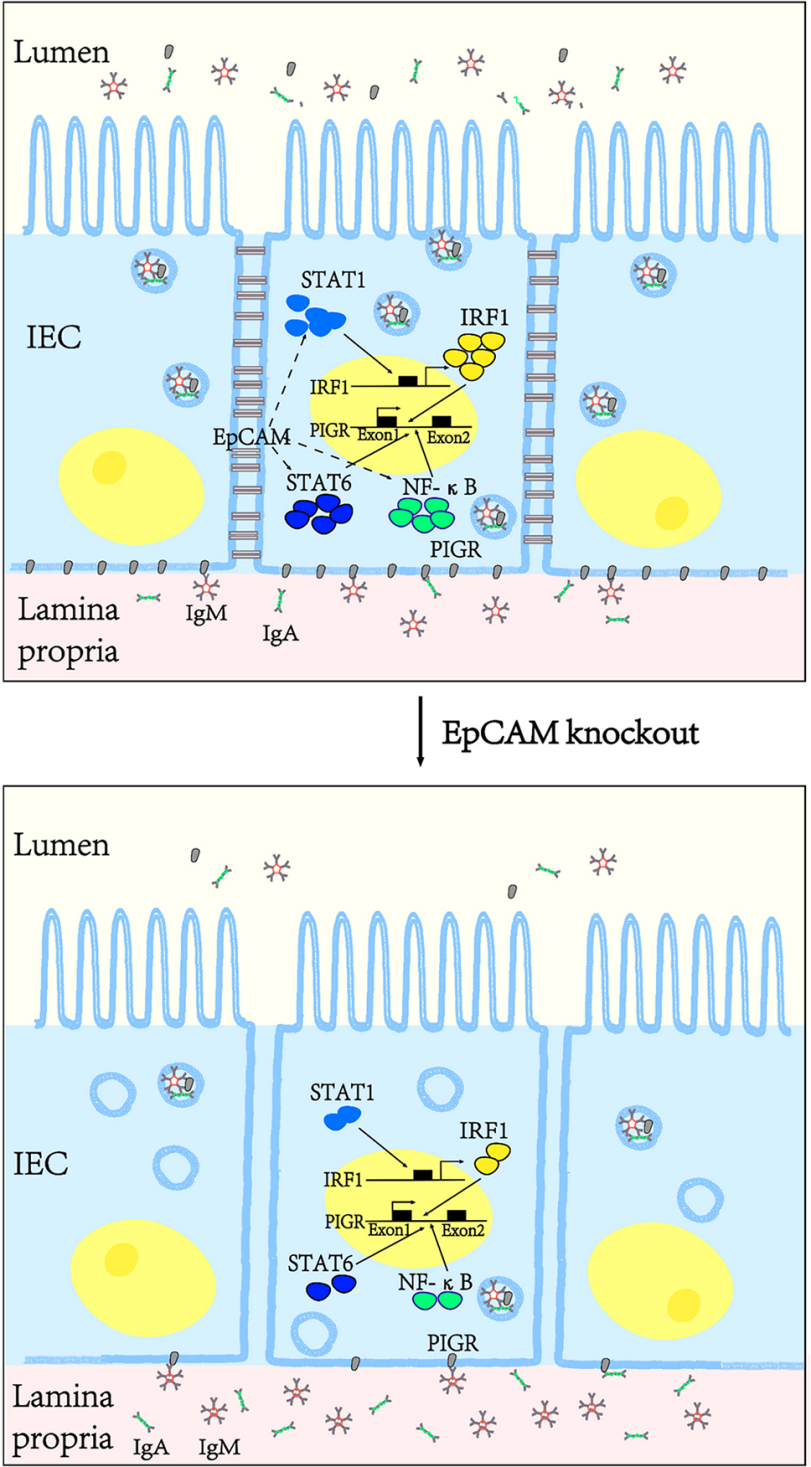
EpCAM deficiency induced the inflammation in the intestines of mice *via* downregulating the expression of pIgR in the intestinal epithelial cells. EpCAM deficiency caused the decrease of the expression and activation of the transcriptional factors including STAT1, STAT6, NF-κB and IRF1, then caused the downregulation of the expression of pIgR in the intestinal epithelial cells, so IgA and IgM which were produced by the plasma cells under the intestinal epithelium could not be transported into the lumen of the intestines to remove pathogens there and then the inflammation occurred in the intestines of these mice.

In conclusion, EpCAM is essential to maintaining the immune homeostasis of intestines *via* keeping the normal level of PIGR in the intestinal epithelium. EpCAM deficiency induced the reductions of the expression and activation of transcriptional factors including STAT1, STAT6, NF-κB and IRF1, then caused the downregulation of pIgR gene in the intestinal epithelium, so IgA and IgM which were produced by plasma cells under the intestinal epithelium could not be transported into the lumen of intestines to remove pathogens there and then the damage of intestinal epithelium occurred because of the breakdown of the intestinal immune homeostasis (**Figure 7**).

## Data Availability Statement

The original contributions presented in the study are included in the article/**Supplementary Material**. Further inquiries can be directed to the corresponding authors.

## Ethics Statement

The animal study was reviewed and approved by the Experimental Animal Ethics Committee of Guangdong Pharmaceutical University.

## Author Contributions

ZL, YY, and JG designed the project. WL and YN maintained the animal models. WL, YN, YY, GC, LH, HW, YL, LC, QH, HR, SY, QS, and FT performed the morphological, biochemical and molecular experiments. WL, YN, and YY created the figures and tables. YY and ZL wrote the draft of the manuscript. All authors had read and approved the final manuscript. We stated that the 4 co-first authors were determined alphabetically according their family names.

## Funding

This work was supported by the National Natural Science Foundation of China (No. 82171855, No. 31671520, No. 81830113, No. 81803912); National key R&D plan “Research on modernization of traditional Chinese medicine” (2018YFC1704200); Major basic and applied basic research projects of Guangdong Province of China (2019B030302005); the Guangdong Basic and Applied Basic Research Foundation (2021A1515012383); the Opening Foundation of the Key Laboratory of Regenerative Biology, Guangzhou Institutes of Biomedicine and Health, Chinese Academy of Sciences (KLRB201807); the Science and Technology Planning Project of Guangzhou City (No. 201803010069); and the Science and Technology Project of Yue-Xiu District of Guangzhou (No. 2018-WS-011). We thank Miss Lanxiang Yang and Miss Yanyan Liu from Guangdong Pharmaceutical University for their technical assistance.

## Conflict of Interest

The authors declare that the research was conducted in the absence of any commercial or financial relationships that could be construed as a potential conflict of interest.

## Publisher’s Note

All claims expressed in this article are solely those of the authors and do not necessarily represent those of their affiliated organizations, or those of the publisher, the editors and the reviewers. Any product that may be evaluated in this article, or claim that may be made by its manufacturer, is not guaranteed or endorsed by the publisher.

## Supplementary Material

Supplementary Figure 1 The morphology and histology of the intestines of EpCAM mutant mice at P0 stage. **(A)** A littermate of P0 pups from one pair of EpCAM^+/-^ parental mice. **(B)** The images of intestines from WT, EpCAM^+/-^ and EpCAM^-/-^ pups at P0 stage. **(C)** Graph showed the relative intestinal lengths of WT and EpCAM^-/-^ pups at P0 and P3 stages, respectively (n=6). **(D)** Images of H&E staining of duodenum, jejunum, ileum and colon from WT and EpCAM^-/-^ pups at P0 stage. **(E)** Images of the immuofluorescence staining of Claudin-7 in sections of jejunum from WT and EpCAM^-/-^ embryos at E18.5 stage. **p* < 0.05, ***p* < 0.01, as determined by Mann–Whitney U test. All error bars represent SDs.

Supplementary Figure 2 The upregulation of genes related to inflammatory factors and the infiltration of inflammatory cells in the intestines of EpCAM^-/-^ P0 pups. **(A, B)** Graphs showed the relative mRNA expression levels of TNF-α, IL-1β, IL-4, IL-6, IL-8rb, COX-2, MIP-2 and MCP-1 in the (A) small and (B) large intestines from WT and EpCAM^-/-^ pups at P0 stage (n=6). **(C, D)** qPCR results of F4/80, Ly6d and Ly6g from the **(C)** small and **(D)** large intestines of WT and EpCAM^-/-^ pups at P0 stage (n=6). **p* < 0.05, ***p* < 0.01, as determined by Mann–Whitney U test. All error bars represent SDs.

Supplementary Figure 3 The changes of the expression of IgA and IgM related genes and the localization of IgA proteins in the intestines of EpCAM knockout mice. **(A, B)** Graphs showed the relative mRNA expression levels of (A) Igha and **(B)** Ighm in the small and large intestines from WT and EpCAM^-/-^ mice at E18.5, P0 and P3 stages, respectively (n=6). **(C)** The images of the immuofluorescence staining of IgA in sections of jejunum and colon from WT and EpCAM^-/-^ mice at E18.5 and P3 stages, respectively (n=3). **p* < 0.05, ****p* < 0.001, as determined by Mann–Whitney U test. All error bars represent SDs.

Supplementary Figure 4 The changes of signaling pathways that regulate the expression of pIgR in the epithelial cells of large intestines from EpCAM knockout mice. **(A)** The relative mRNA expression levels of Irf1, Stat1, Stat6, p50, p65, p100, Relb, Tlr2, Tlr4, Myd88, IFNγ, Jak1 and Jak3 in the large intestines from WT and EpCAM^-/-^ embryos at E18.5 stage (n=6). **(B)** Graph showed the qPCR results of Irf1, Stat1, Stat6, p50, p65, p100, Relb, Tlr2, Tlr4, Myd88, IFNγ, Jak1 and Jak3 in the large intestines from WT and EpCAM^-/-^ mice at P3 stage (n=6). **(C, D)** Western blot results of IRF1, p-STAT1, STAT1, p-JAK2, JAK2, MYD88, p-p65, p65, p-STAT6 and STAT6 in the large intestines from WT and EpCAM^-/-^ mice at **(C)** E18.5 and **(D)** P3 stages, respectively (n=3). **p* < 0.05, ***p* < 0.01, as determined by Mann–Whitney U test. All error bars represent SDs.

Supplementary Figure 5 EpCAM deficiency caused the changes of signaling pathways which regulate the expression of pIgR in the intestinal epithelium of mice at P0 stage. **(A, B)** Graphs showed the qPCR results of Irf1, Stat1, Stat6, p50, p65, p100, Relb, Tlr2, Tlr4, Myd88, IFNγ, Jak1 and Jak3 in the **(A)** small and **(B)** large intestines from WT and EpCAM^-/-^ mice at P0 stage, respectively (n=6). **(C, D)** Western blot results of IRF1, p-STAT1, STAT1, p-JAK2, JAK2, MYD88, p-p65, p65, p-STAT6 and STAT6 in the **(C)** small and **(D)** large intestines from WT and EpCAM^-/-^ mice at P0 stage, respectively (n=3). **p* < 0.05, ***p* < 0.01, as determined by Mann–Whitney U test. All error bars represent SDs.
