# Supplementary figures and images for: EpCAM Is Essential to Maintaining the Immune Homeostasis of Intestines via Keeping the Expression of pIgR in the Intestinal Epithelium of Mice
Source: Front Immunol. 2022 Apr 13;13:843378. doi: 10.3389/fimmu.2022.843378 (PMC9043958; doi:10.3389/fimmu.2022.843378)

Figure.S1

A

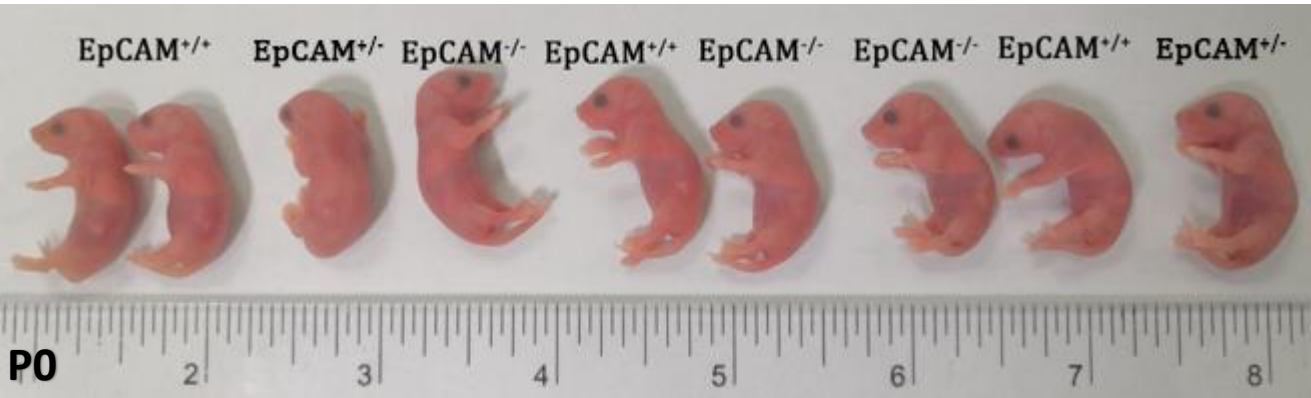

B

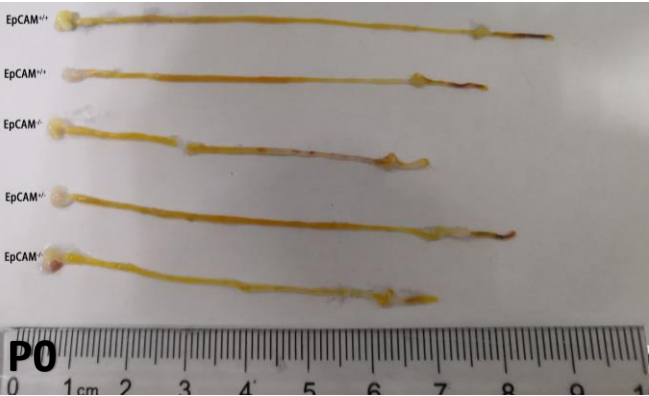

C

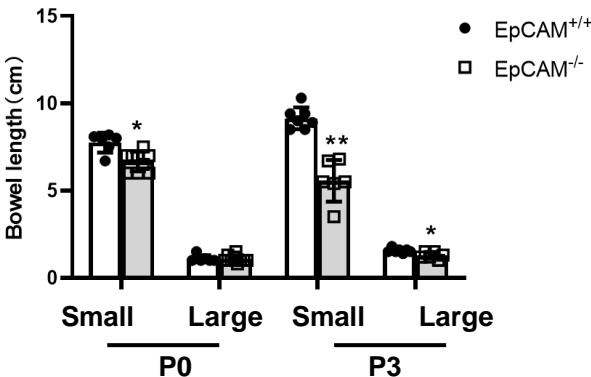

D

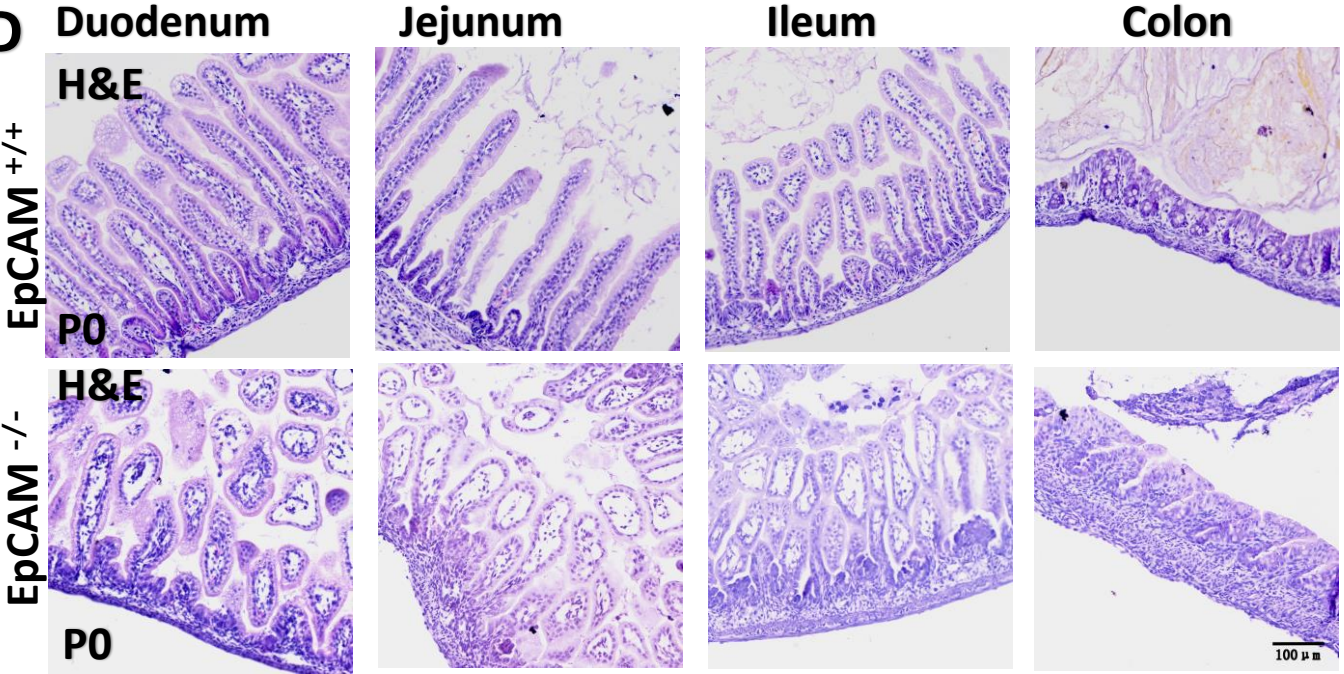

E

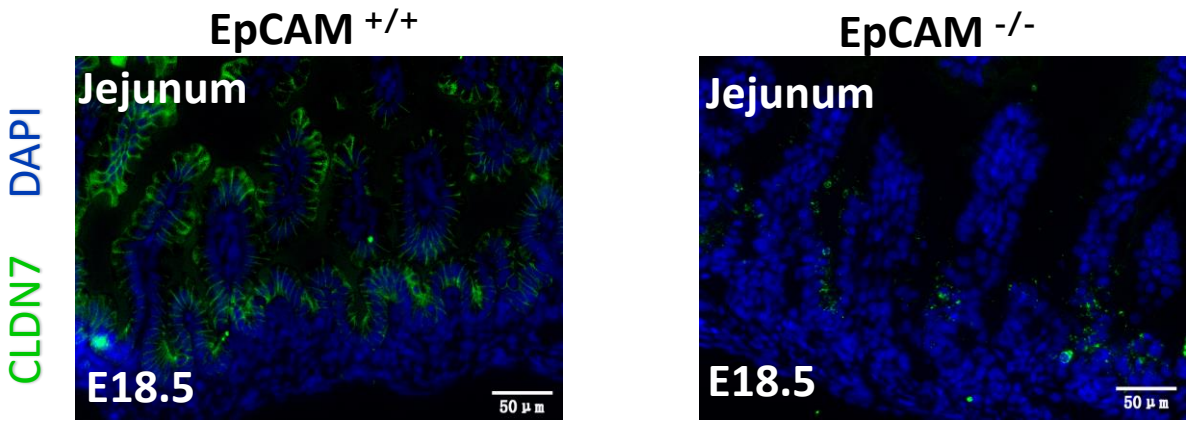

Figure.S2

A

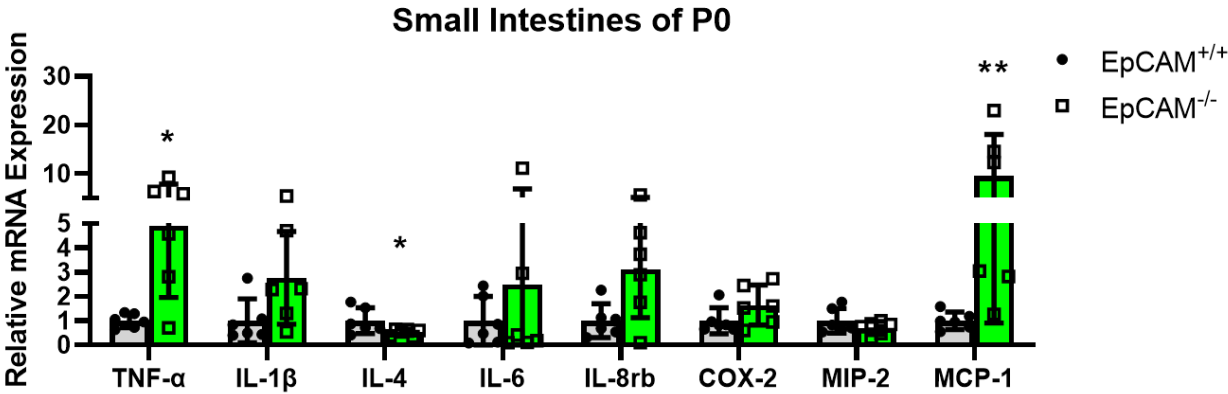

B

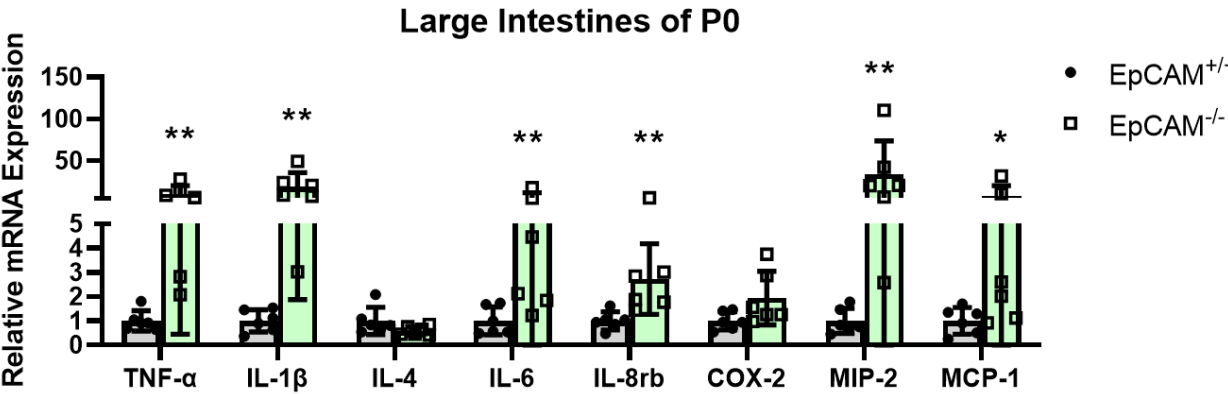

C

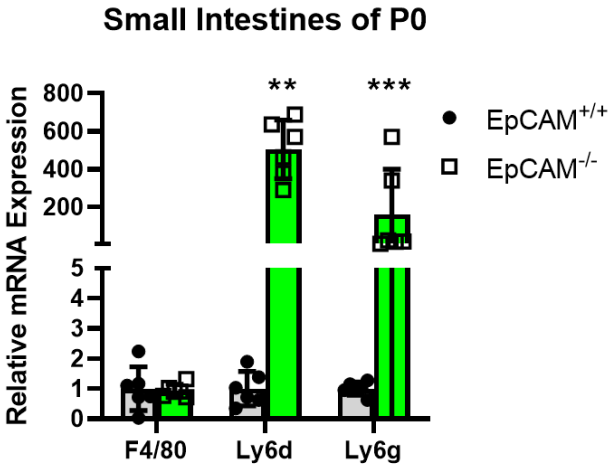

D

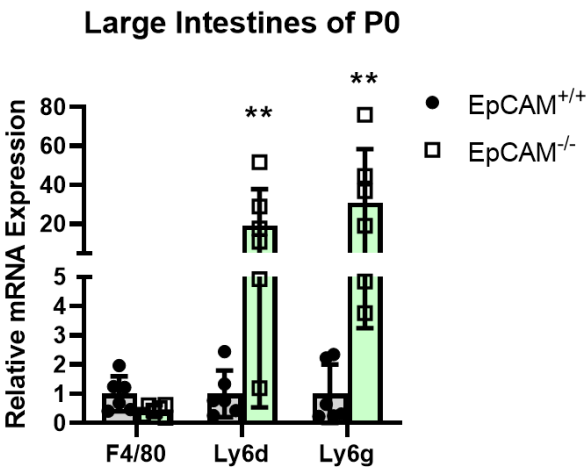

Figure.S3

A

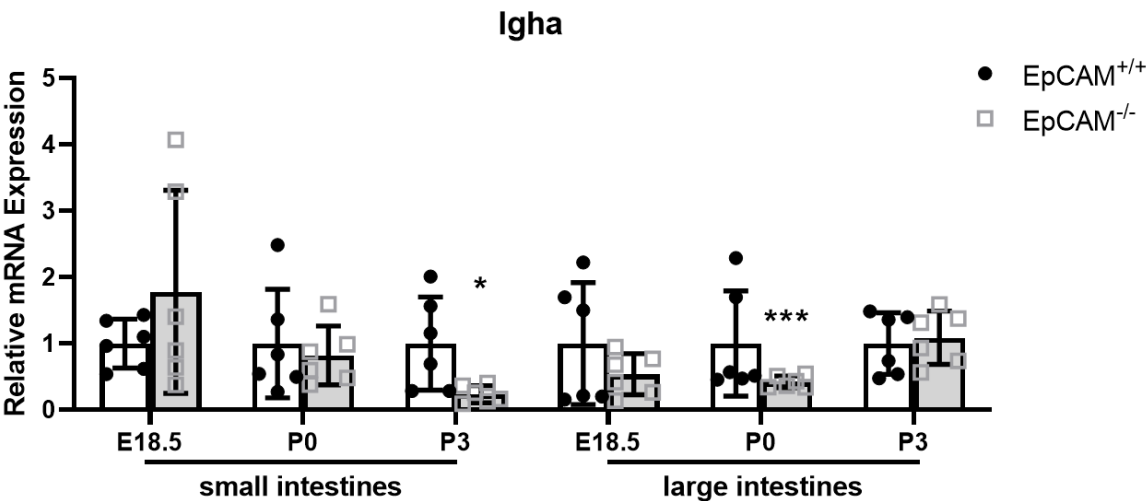

B

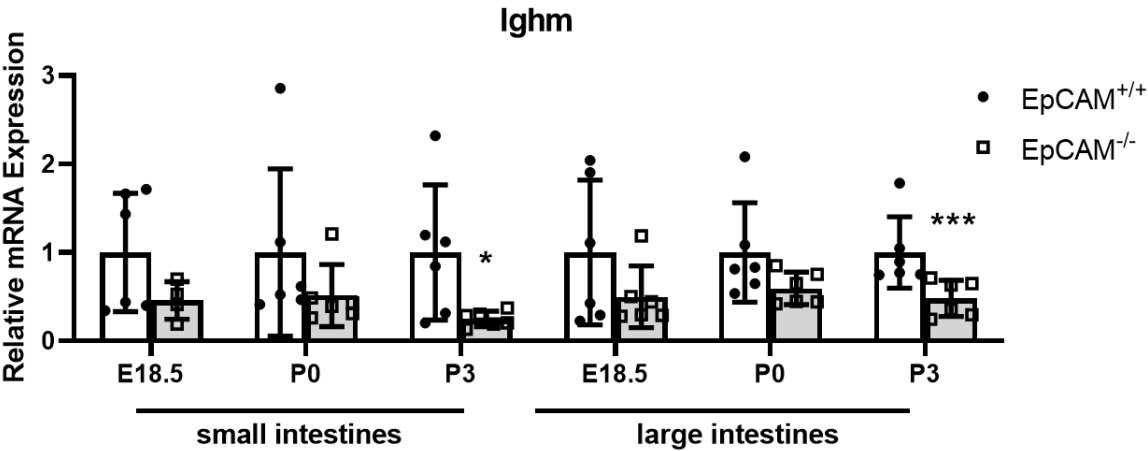

C

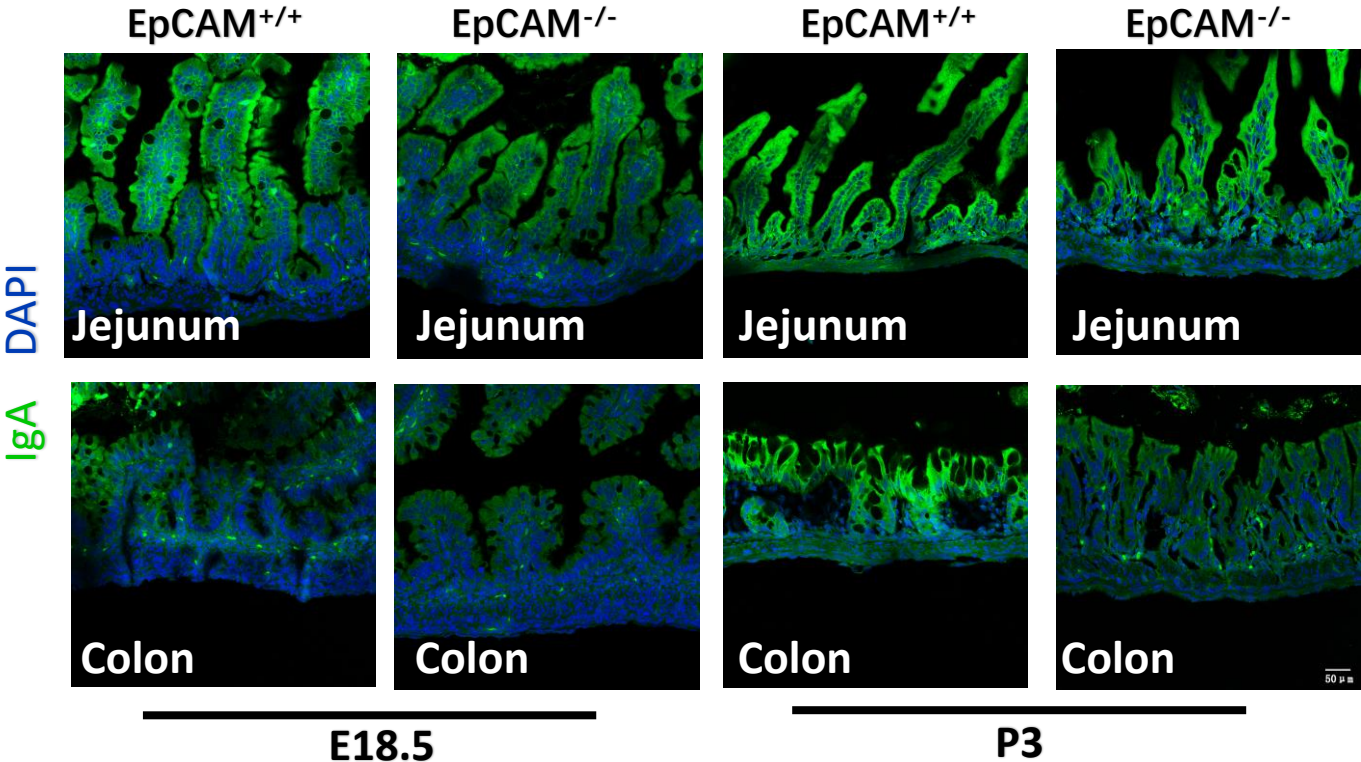

Figure.S4

A

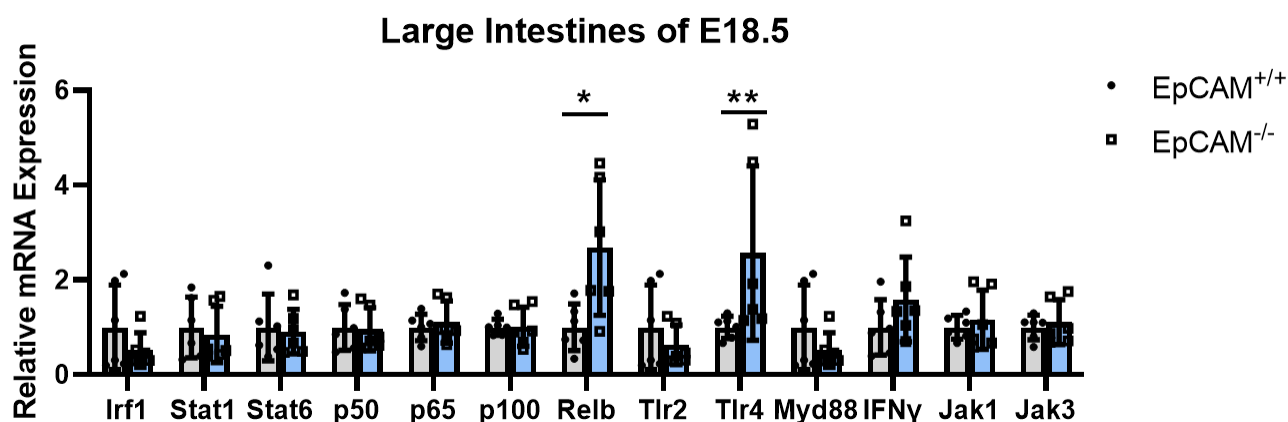

B

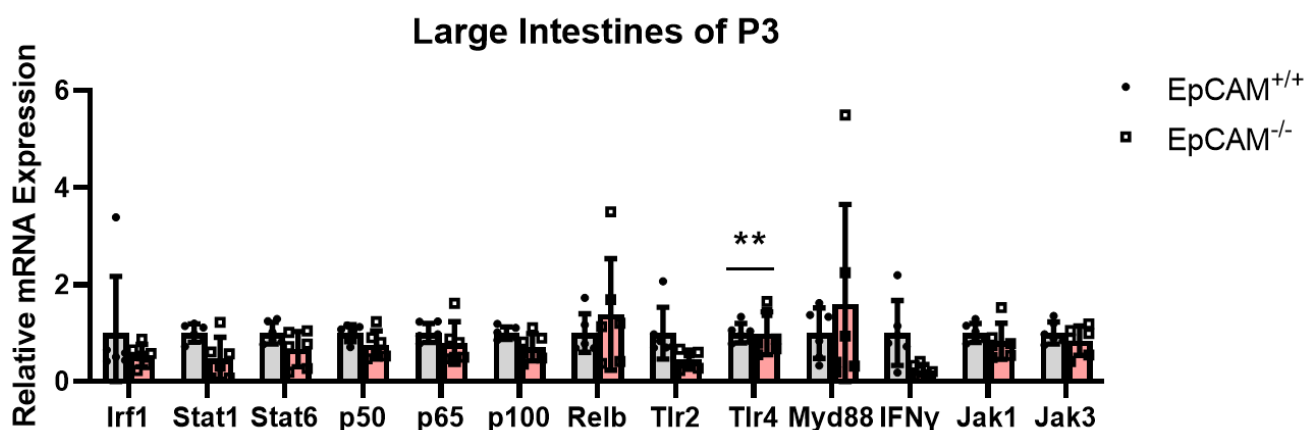

C

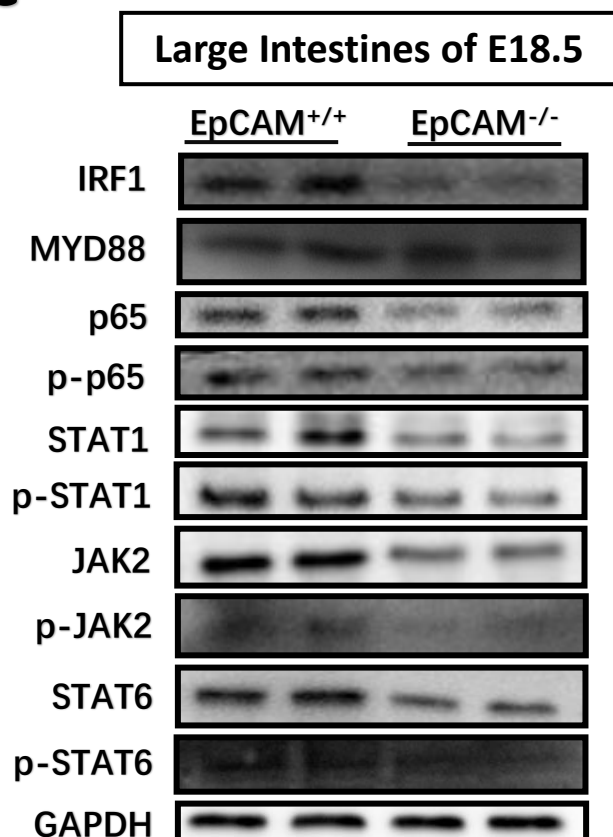

D

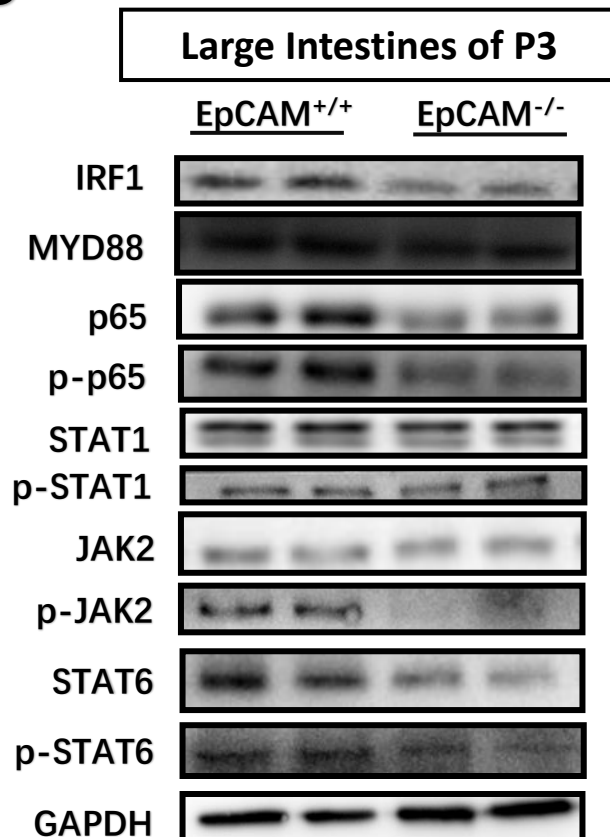

Figure.S5

A

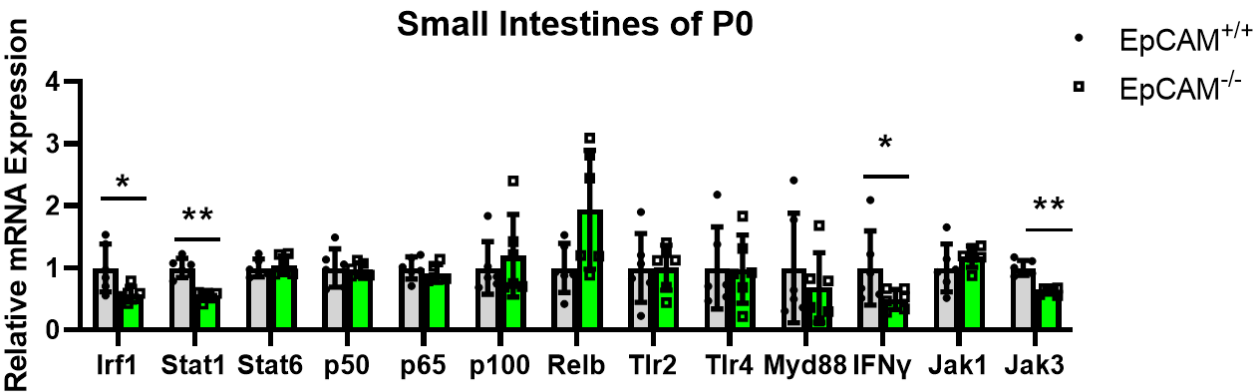

B

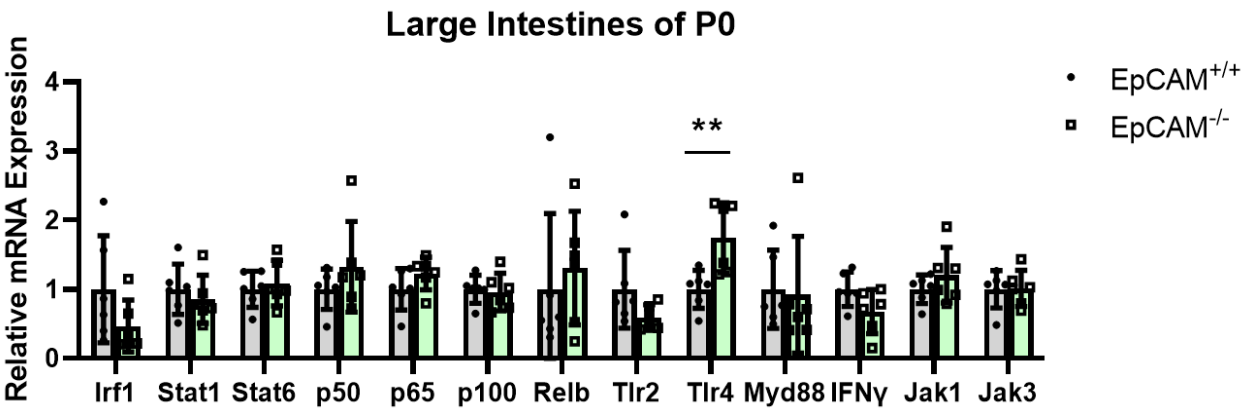

C

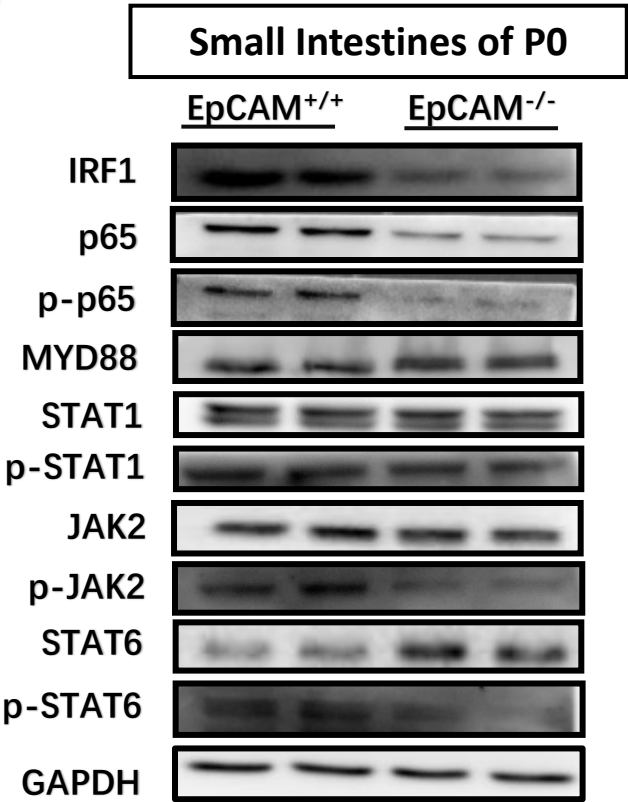

D

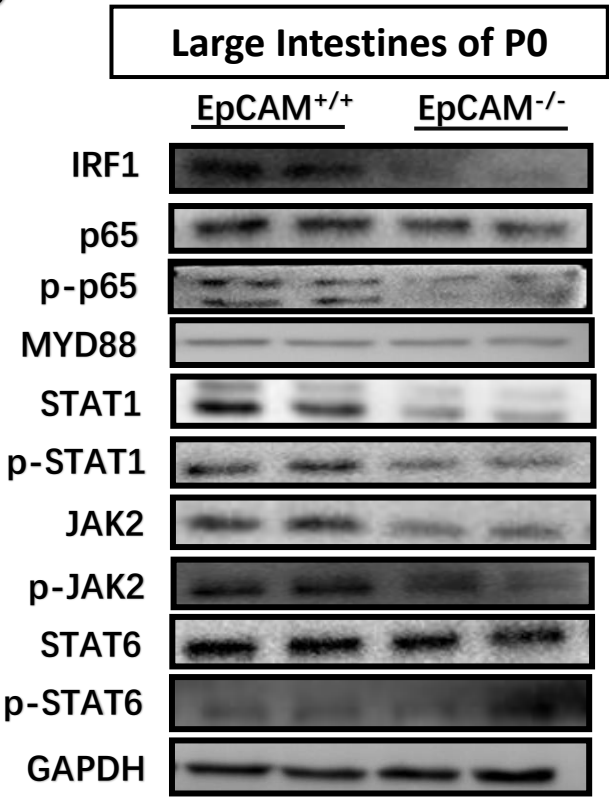

Supplement: Supplementary Figure 1 — The morphology and histology of the intestines of EpCAM mutant mice at P0 stage. (A) A littermate of P0 pups from one pair of EpCAM+/- parental mice. (B) The images of intestines from WT, EpCAM+/- and EpCAM-/- pups at P0 stage. (C) Graph showed the relative intestinal lengths of WT and EpCAM-/- pups at P0 and P3 stages, respectively (n=6). (D) Images of H&E staining of duodenum, jejunum, ileum and colon from WT and EpCAM-/- pups at P0 stage. (E) Images of the immuofluorescence staining of Claudin-7 in sections of jejunum from WT and EpCAM-/- embryos at E18.5 stage. *p < 0.05, **p < 0.01, as determined by Mann–Whitney U test. All error bars represent SDs. [file DataSheet_1.pdf]
