# Supplementary material for: EpCAM Is Essential to Maintaining the Immune Homeostasis of Intestines via Keeping the Expression of pIgR in the Intestinal Epithelium of Mice
Source: Front Immunol. 2022 Apr 13;13:843378. doi: 10.3389/fimmu.2022.843378 (PMC9043958; doi:10.3389/fimmu.2022.843378)
Supplement: Supplementary file 2 [file Table_1.docx]

Table S1. Primer sequences of genes for qPCR

Table S2. Information of antibodies used for western blot

| Antibodies | Species | Cat. No. | Company | Dilutions |
| --- | --- | --- | --- | --- |
| Anti-Mouse | donkey | ab205724 | Abcam | 1/2000 |
| Anti-Rat | donkey | ab102182 | Abcam | 1/2000 |
| Anti-Rabbit | goat | SA00001-2 | Proteintech | 1/2000 |
| GAPDH | rabbit | 14C10 | Cell Signaling Technology | 1/1000 |
| IRF1 | rabbit | 8478 | Cell Signaling Technology | 1/1000 |
| JAK2 | rabbit | 3230 | Cell Signaling Technology | 1/1000 |
| JNK | rabbit | 9252 | Cell Signaling Technology | 1/1000 |
| MMP3 | rabbit | ab63853 | Abcam | 1/1000 |
| MMP7 | rabbit | ab38996 | Abcam | 1/1000 |
| MMP8 | rabbit | ab81286 | Abcam | 1/1000 |
| p38 | rabbit | ab38238 | Abcam | 1/1000 |
| p65 | rabbit | ab32536 | Abcam | 1/1000 |
| p-p65 | rabbit | 3031 | Cell Signaling Technology | 1/1000 |
| p-ERK1/2 | rabbit | 9101 | Cell Signaling Technology | 1/1000 |
| pIgR | rat | ab170321 | Abcam | 1/1000 |
| p-JAK2 | rabbit | 3776 | Cell Signaling Technology | 1/1000 |
| p-JNK | mice | 9255 | Cell Signaling Technology | 1/1000 |
| p-p38 | rabbit | ab38238 | Abcam | 1/1000 |
| p-STAT1 | rabbit | 9167 | Cell Signaling Technology | 1/1000 |
| p-STAT6 | rabbit | 56554 | Cell Signaling Technology | 1/1000 |
| STAT1 | rabbit | 14994 | Cell Signaling Technology | 1/1000 |
| STAT6 | rabbit | 5397 | Cell Signaling Technology | 1/1000 |
| TLR2 | mice | ab16894 | Abcam | 1/1000 |
| TLR4 | mice | ab22048 | Cell Signaling Technology | 1/1000 |
